# Bilateral Subdural Hematomas Without Midline Shift: A Case Report

**DOI:** 10.7759/cureus.72220

**Published:** 2024-10-23

**Authors:** Juhi Patel, Nicholas D Luke, Mehrdad Alaie

**Affiliations:** 1 Emergency Medicine, St. Barnabas Hospital Health System, New York City, USA

**Keywords:** glasgow coma scale, glasgow coma scale score, midline shift, neurological outcomes, neurosurgery, subdural hematoma

## Abstract

Subdural hematomas are blood collections between the dura mater and the arachnoid mater of the meninges that can rapidly progress into surgical emergencies. Commonly seen in the elderly and alcoholic demographics, the accumulation of blood can cause midline shifts and brain herniation. The standard practice of treatment may include either a craniotomy or burr hole trephination. Subdural hematomas may be acute or chronic and be unilateral or bilateral; predicting mortality rate can be done based on the Glasgow Coma Scale (GCS) score, age of the patient, hemodynamic stability, neurological deficits, and radiological findings such as location of the bleed and measurement of midline shift. We present a 93-year-old male patient who was found to have acute-on-chronic bilateral subdural hematomas without a midline shift a week after he had a fall at home. The patient was neurologically intact and not in respiratory distress. Bilateral burr hole trephination was used to evacuate the hematomas, and the patient was closely followed up postoperatively and did not have any complications and was discharged. This case provides an interesting look at managing bilateral subdural hematomas without midline shift or neurological deficits and stable hemodynamics.

## Introduction

Within the skull are three separate layers of tissues (pia mater, arachnoid mater, and dura mater) that protect the brain. These three layers, also known as the meninges, are all potential spaces for blood to collect [1]. In particular, the space between the dura mater and the arachnoid mater of the meninges can lead to the formation of a subdural hematoma. The mechanism of injury of a subdural hematoma typically consists of a traumatic force applied to the head that causes changing velocities of the contents within the skull [1]. The prognosis of a subdural hematoma depends on the amount of blood accumulated within the subdural space. A small bleed can resolve independently, whereas a moderate to significant bleed can be due to sheared bridging veins after swift velocity changes, eventually leading to herniation of cerebral structures [2]. The pressure behind a worsening subdural hematoma can cause a midline shift in the brain, lowering the Glasgow Coma Scale (GCS) score (altered mental status) and eventually leading to Cushing’s reflex, with bradycardia, hypertension, and irregular breathing patterns. 

## Case presentation

We present a 93-year-old male patient with a medical history of vascular dementia, heart failure with reduced ejection fraction, coronary artery disease with stent placement, diabetes mellitus, hypertension, chronic kidney disease, and hypothyroidism who presented to the emergency room after a mechanical fall about a week ago. The patient fell in the shower with a review of systems positive for loss of consciousness, dizziness, and nausea with non-bloody and non-bilious vomiting. The patient's home health aide stated he usually is ambulatory with a walker, compliant with his home medications, and awake, alert, and oriented to self, time, and place at baseline. The patient did not want to come to the emergency department initially, but he developed left-sided chest pain from the point of impact after the fall, which was worsening in severity. The vital signs in triage were the following: temperature of 36.4 °C (97.5 °F), a heart rate of 71 beats per minute, respiratory rate of 19 breaths per minute, blood pressure of 155/76 millimeters of mercury, and oxygen saturation of 100% on room air. 

The physical examination was remarkable for point tenderness in the left lower rib area with ecchymosis. The GCS score was 15 during the initial assessment. There were no signs of acute trauma on the head, ears, nose, and throat. The patient did not have step-offs or midline tenderness on the cervical, thoracic, and lumbar spines. The neurological exam was also unremarkable; the patient was alert, cranial nerves were intact, there were no sensory or coordination deficits, and the gait was intact. 

**Figure 1:**
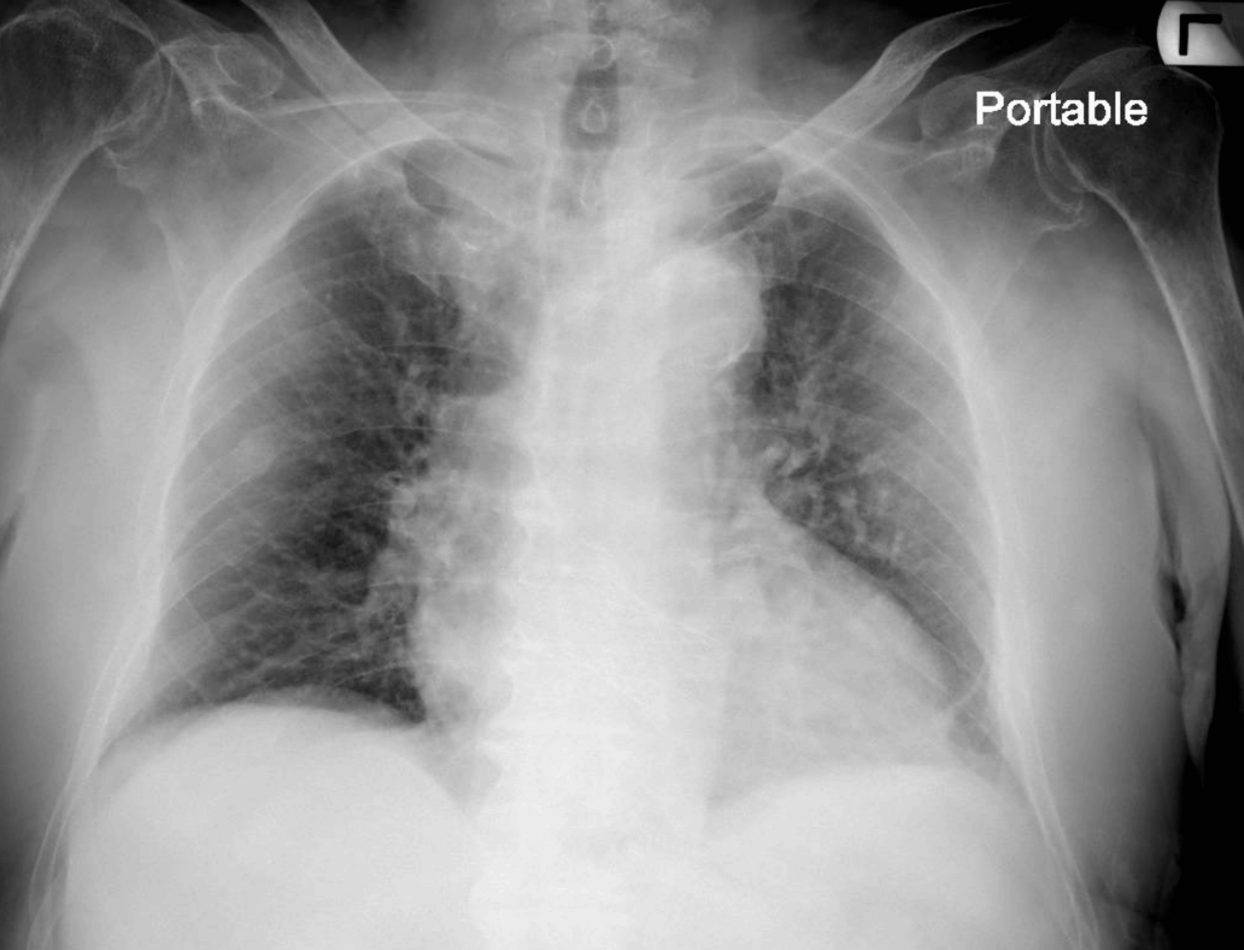
Portable one-view chest X-ray of the patient demonstrating a healed right-sided rib fracture likely from prior trauma, mild congestive heart failure, and no significant acute traumatic injury detected.

**Figure 2:**
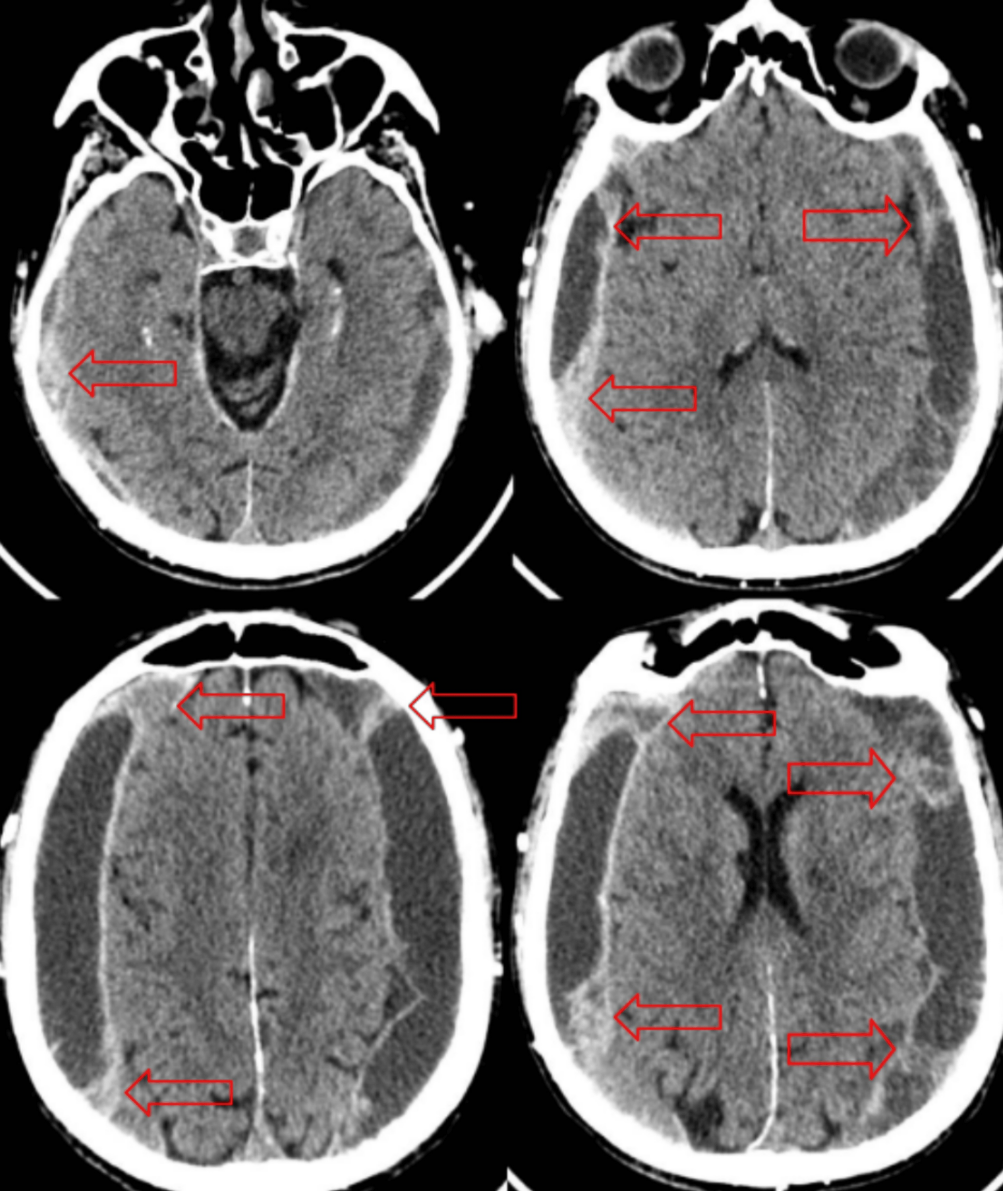
Several slides of the initial computed tomography scan of the head depicting acute on chronic subdural hematomas (red arrows) with mass effect on the adjacent brain parenchyma and bilateral lateral ventricles. There was no evidence of a significant midline shift or large acute territorial infarct.

The medical workup of this patient consisted of a focused assessment with sonography for trauma (no positive findings) and an x-ray of the chest, which showed an old healed right rib fracture and mild congestive heart failure with no acute traumatic findings (Figure 1), and computed tomography (CT) scans of the brain (Figure 2) and cervical spine which showed significant bilateral acute on chronic subdural hematomas, measuring up to 2.0 cm in maximal thickness on the right and 2.1 cm in maximal thickness on the left, demonstrating mass effect on the adjacent brain parenchyma and bilateral lateral ventricles without midline shift, and diffuse osteopenia with no acute fractures/step-offs, respectively. An electrocardiogram was also performed; it demonstrated normal sinus rhythm with no signs of ischemia or cerebral T-waves.

The blood work demonstrated a negative troponin I level, a brain natriuretic peptide level of 232 ng/ml, an activated partial thromboplastin time of 22.5 seconds, a prothrombin time of 10.5 seconds, an international normalized ratio of 1.02, a sodium level of 139 mEq/L, a potassium level of 5.2 mEq/L, a chloride level of 103 meq/L, a bicarbonate level of 23 meq/L, an anion gap of 13 meq/L, a creatinine level of 2.4 mg/dl, a glucose level of 229 mg/dl, a venous pH of 7.466 units, a venous partial pressure of carbon dioxide (pCO_2_) of 37.9 mmHg, a venous lactate level of 2.1 mmol/L, a white blood cell count of 8.8x10^3/uL, a hemoglobin level of 10.1 g/dL, the hematocrit was 32.6%, and a platelet level of 289x10^3/uL. 

The initial CT scan of the head reported interval development of large bilateral acute on chronic subdural hematomas, demonstrating the mass effect on the adjacent brain parenchyma and bilateral lateral ventricles; no other evidence of acute intracranial hemorrhage, significant midline shift, or large acute territorial infarct. The basilar cisterns appeared patent-stable age-related atrophy, with mild chronic microvascular ischemic changes and multiple remote lacunar infarcts. Stable intracranial atherosclerosis were also seen (Figure 2). The neurosurgery team was consulted, and they recommended admission to the surgical critical care unit, give Keppra 500 mg twice a day, and prepare the patient for a bilateral burr hole procedure. A CT head scan was repeated six hours later, demonstrating no changes to the size of the bilateral subdural hematomas or the mass effect on the bilateral frontal convexities. After admission, the patient had a bilateral burr hole procedure on day two and was monitored closely postoperatively for nine days in total. The burr hole procedure was performed in tandem fashion, with the same steps being performed consecutively. The procedure was initiated on the right side and then on the left. The procedure took place on the parietal bosses bilaterally. The irrigation of the bilateral hematomas was performed with copious amounts of body-temperature sterile normal saline solution.

**Figure 3:**
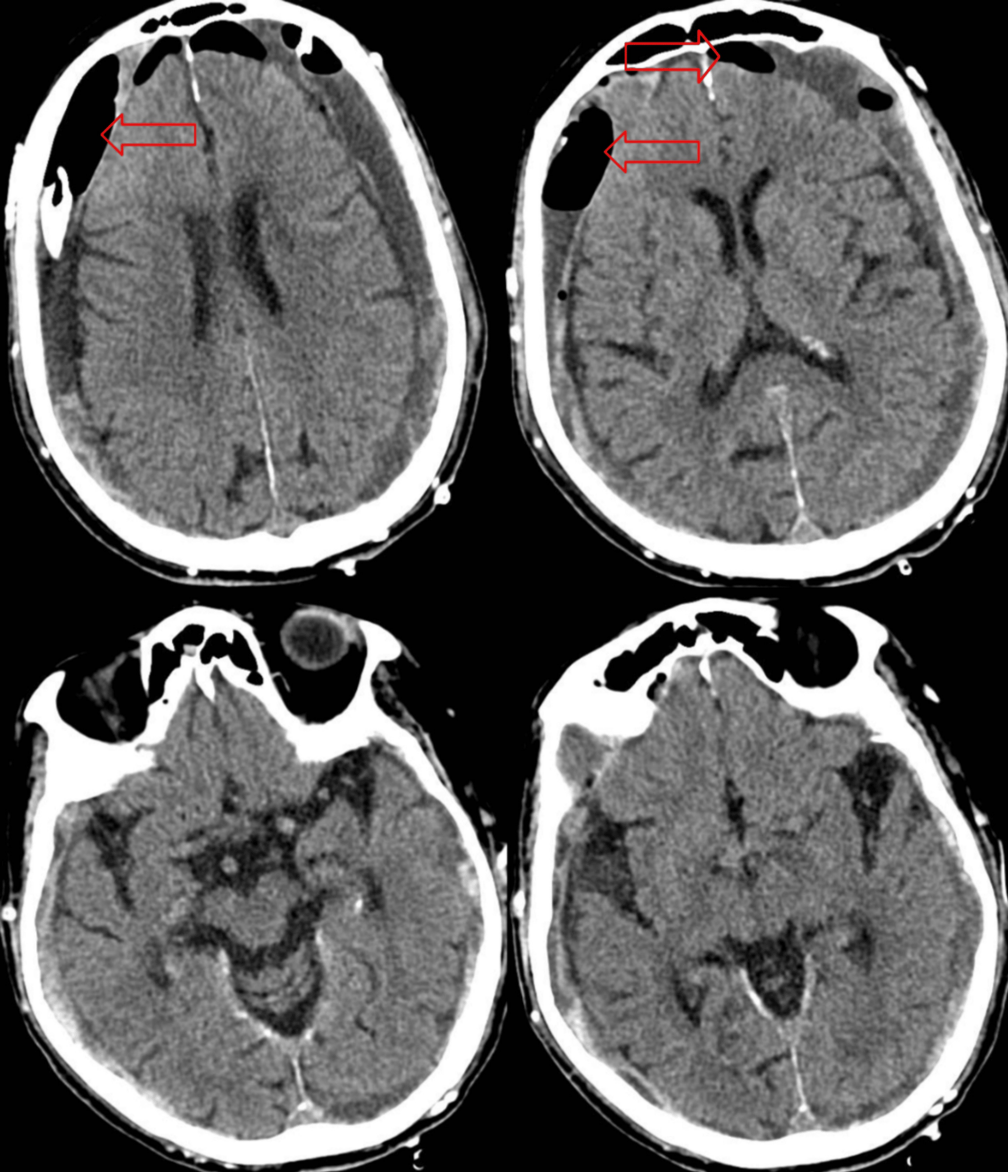
Several slides from the postoperative CT head image study depicting interval bilateral small parietal craniotomies with the placement of bilateral subdural drains since the prior examination with partial evacuation of the significant acute on chronic subdural hematomas, now containing pneumocephalus (red arrows). There is no midline shift. No acute parenchymal hemorrhage. Stable appearance of the brain parenchyma and stable size and configuration of the ventricles and sulci. The basal cisterns are patent.

Postoperatively, the repeat CT head scan depicted interval bilateral small parietal craniotomies and placement of bilateral subdural drains since the prior examination with the partial evacuation of the large acute on chronic subdural hematomas, now containing pneumocephalus. There was no midline shift. No acute parenchymal hemorrhage was noted. There was a stable appearance of the brain parenchyma and stable size and configuration of the ventricles and sulci. Basal cisterns were patent (Figure 3). Postoperatively, the patient was doing well with well-healing surgical sites. The physical exam was unremarkable, with no new neurological deficits, and the patient was awake, alert, and oriented to self, place, and time. 

## Discussion

Acute-on-chronic subdural hematomas are commonly seen in alcoholics and the elderly population, especially in the setting of trauma, which both groups are prone to. Interestingly, cases reported typically have some degree of midline shift associated with the subdural hematoma. In a study by Lee et al., all the patients had midline shifts ranging from 8.8 millimeters to 26.5 millimeters [3]. Our patient had no apparent midline shift seen on CT imaging before and after surgical intervention. In Lee et al.’s study, the studied patient group had symptoms such as hemiparesis and headaches, and it is hypothesized that the brain atrophy seen in elderly patients may have delayed symptomatology [3]. The atrophy seen in aging patients may allow blood to accumulate within the atrophied spaces undetected until the hematoma is large enough to cause a midline shift, and then, in turn, symptoms may start to appear as a delayed presentation [3]. Initially, our patient had a positive review of systems for an unsteady gait, dizziness, nausea, and vomiting. His initial presentation was concerning possible acute intracranial pathology before imaging; however, he was able to protect his airway and was mentating well during the initial visit to the emergency department. 

Our presenting patient was managed with a bilateral burr hole procedure to evacuate the acute blood collection. While the procedure was performed successfully and the patient retained his mental status and had good neurological outcomes, it is vital to raise awareness of the complications of neurosurgical intervention. Such complications can include cutaneous and intracranial infections, acute bleeding, parenchymal injury secondary to misplacement, midline shift if one side is drained faster than the other, and brain herniation. Another factor to consider is which side to drain. A neurosurgeon may drain the dominant side of the brain, usually the left side, first to preserve as much neurological function as possible. Other factors to consider include anatomy and the presence of midline shift. If one subdural hematoma is causing more midline shift than the other, it may be reasonable for the neurosurgeon to drain that side first. In our presenting patient, there was no midline shift associated with the acute-on-chronic bilateral subdural hematomas, so this factor had less influence on the sequence of drainage. In one case report, a patient complaining of severe headache was found to have chronic bilateral subdural hematomas [4]. The patient was taken to the operating room for burr hole trephination but rapidly decompensated shortly after the procedure [4]. The patient had a rapid duret hemorrhage in the brainstem and downward herniation after emergent surgical intervention, and the patient expired despite efforts to reduce his intracranial pressure. Although the patient underwent surgical intervention, the delay in seeking medical attention (about three days of symptoms before the hospital visit) may have led to this rare, poor outcome of fatal brain herniation. In the case of our presenting patient, he delayed medical attention for about a week. Although he did not have any postoperative complications, it is crucial to monitor the patient and be aware of any changes in mental status or respiratory distress. If nonsurgical management is required in the setting of increased intracranial pressure, the utilization of mannitol, corticosteroids, and tranexamic acid (increased pressure with brain hemorrhage) has been considered. However, data is limited in the setting of bilateral chronic subdural hematomas in particular [4]. 

Predicting mortality rates for patients with intracranial bleeding can be determined by several factors. Pastor et al. studied the factors that predict the 30-day mortality rate in patients with traumatic subdural hematomas [5]. Pastor et al. determined that a Glasgow coma scale score with a cut-off value of ≤12; secondary brain injuries such as intracranial hypertension, brain herniation, and brain swelling; associated cranial lesions such as intraparenchymal hematoma, cranial fracture, and midline shift with a cut-off value >7 mm [5]. Pastor et al. also determined that, in a multivariate analysis, GCS ≤12 and midline shift >7 mm were independent predictive factors for 30-day mortality after head trauma [5]. Our presenting patient had a traumatic bilateral subdural hematoma; however, he did not have a midline shift on imaging. His GCS was 15 throughout the hospital stay. Predicting mortality in our patient is unique because the patient had no midline shift and underwent surgical intervention; however, he was neurologically intact and had a GCS of 15. The risk of mortality in our patient is still present due to neurosurgical intervention. With this in mind, clinicians should closely monitor the patient's vital signs and neurological status postoperatively.

## Conclusions

Subdural hematomas are commonly seen in alcoholics and the elderly, especially in the setting of trauma. The accumulation of blood between the dura mater and the brain can lead to emergent surgical interventions. Interventions typically involve neurosurgery to relieve the pressure created by the accumulation of blood between the meninges and the brain. Some unique presentations of subdural hematomas may not have features such as midline shift or hemodynamic instability. Monitoring the patient closely for rapid decompensation is vital, as predicting mortality rates at any point during the hospital visit may be challenging.
